# Spontaneous uterine rupture after abdominal myomectomy at the gestational age of 20 weeks in pregnancy: A case report 

**Published:** 2016-07

**Authors:** Hamideh Pakniat, Nasrin Soofizadeh, Marzieh Beigom Khezri

**Affiliations:** 1 *Department of Obstetrics and Gynecology, Faculty of Medicine, Qazvin* *University of* *Medical Sciences, Qazvin, Iran.*; 2 *Department of Obstetrics and Gynecology, Faculty of Medicine, Kurdistan University of Medical Sciences, Kurdistan, Iran.*; 3 *Department of Anesthesiology, Faculty of Medicine, Qazvin University of Medical Sciences, Qazvin, Iran.*

**Keywords:** *Myomectomy*, *Pregnancy*, *Uterine rupture*

## Abstract

**Background:**

Uterine rupture in pregnancy is rare and often could be life threatening and catastrophic. Myomectomy is one of very common surgeries in gynecology, performed as the vaginal, abdominal and laparoscopic surgeries. Pregnancies occured after abdominal and laparoscopic myomectomy are high risk for uterine rapture.

**Case:**

Patient was a 28 Years old female, pregnant woman at the 20 wks of gestational age with abdominal pain and a history of abdominal myomectomy 6 yrs ago. Uterus was ruptured and fetus in amniotic sac was found in abdominal cavity.

**Conclusion:**

Early diagnosis of uterine rupture after myomectomy can save patients from death.

## Introduction

Uterine rupture during pregnancy involved all uterine wall thickness and uterine serosa. Different factors increase this risk such as: congenital uterine anomalies, multiparty, previous myomectomy, labor induction, uterine trauma and previous uterine scar including (previous cesarean section, abdominal and laparoscopic surgeries, Dilation & curettage (D&C) (1-4). Almost all cases of uterine rupture after myomectomy are occured at 3^rd^ trimester or labor (5). The incidence of uterine rupture is 0.012% in cases without previous scars. But even in high risk group, total incidence rate is low. The rate of total uterine rupture has been reported about 0.07% (1).

Signs and symptoms of uterine rupture are nonspecific leading difficult diagnosis and delay in treatment. Classic signs and symptoms include: fetal distress (FHR abnormalities, decreasing in uterine tone and uterine contraction disappearing, abdominal pain, presenting part station changing, bleeding and shock (6). Braun and colleagues reported the 120 term pregnancies after abdominal myomectomy without uterine rupture and 80% of deliveries had normal vaginal delivery (7). But Garnet and colleagues had 3 casas of uterine rupture in 83 women (4%) with previous myomectomy scar that underwent to elective cesarean section (8). A research in Japan showed no rupture of uterine in 59 patients with laparoscopic myomectomy (9). Only 2 cases of spontaneous uterine rupture are existed before 20 wks of gestational age (GA) in the literature (5, 10). In another study, spontaneous uterine rupture rate at the 33 wks of GA was reported 0.26% (11). Some researchers showed that rupture after laparoscopic myomectomy occurs later and up to 8 yrs after primary surgery (12).

Here we discuss a case of uterine rupture at 20 wks of gestational age of pregnancy with history of previous uterine abdominal myomectomy which is a rare case.

## Case report

Before presentation of the case, informed consent was obtained from the patient. 

This patient was 28 years old female, married 5 years ago, at the GA of 20 wks referred with suddenly diffused abdominal pain. She had a history of infertility for 4 yrs and, appendectomy in 2008 & abdominal myomectomy in 2009 with Pfannestiel incision, which 19 myoma were extracted throughout the uterus and the patient was emphasized that termination of pregnancy should be with cesarean section. She also had a history of hysteroscopy and laparoscopy in 2013 for infertility follow up which was reported a lot of abdominal and pelvic adhesion. Patient had no history of pregnancy and just had an unsuccessful IVF attempt.

This pregnancy was induced by IVF and prenatal care was performed by a specialist. The last visit was done about 2 wks ago and everything was reported normal via sonography, and there was no mention about the exact location of the placenta. On examination, the abdomen was tender in hypogastria and fundal height of uterus was about 20 wks. Observations showed hear beatt of 86 beats per min, and blood pressure (BP) was 90/60 mmHg. Sonography was performed in emergency ward urgently that showed free fluid in coledusac & pelvic fossa, fetus with bradycardia in amniotic sac in abdominal cavity, near the abdominal wall. In hemogram, Hb, HCT and PLT were 10.1 gr /dl & 29.8 & 238000 respectively, and other coagulation tests also were normal.

**Figure 1:**
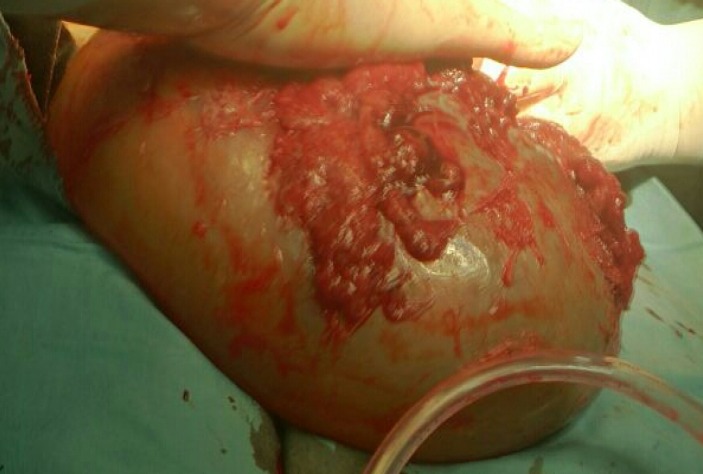
Gestational sac with the fetus.

**Figure 2:**
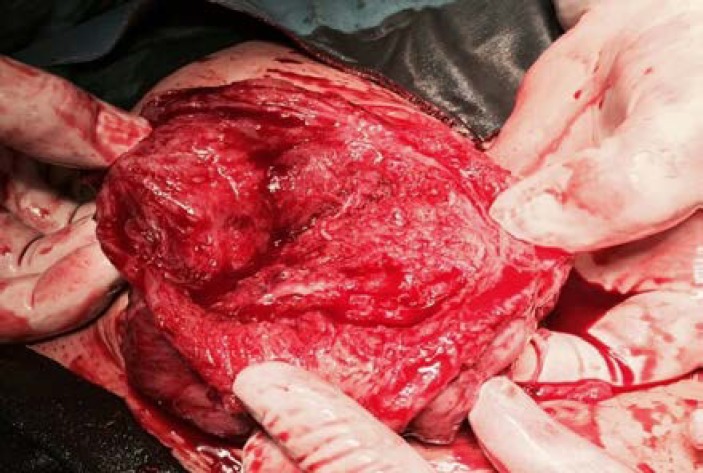
Uterine rupture at the previous myomectomy scar.

After vital sign stability and fixing 2 IV lines and reserving packed cell for transfusion, patient transferred to operation room. An emergency laparotomy was performed immediately with simultaneous ongoing resuscitation. Intraoperative findings revealed about 1000 ml blood in abdominal cavity and between of intestinal loops. Uterus was torn in corneal zone and huge part of gestational sac with fetus and amniotic fluid moved away from tearing zone. Rupture was in uterus fundal and cornea in previous myomectomy scar (Figure 1, 2). During surgery uterus repair was done and 4 units packed cell and 2 units FFP were transfused. She was discharged from hospital with good condition. 

## Discussion

In our patient uterine rupture was occurred at 20 wks of GA that even it is a rare condition after abdominal myomectomy. In this case, only clinical manifestation and history was favored to uterine rupture. She had severe abdominal and nausea and vomiting that are very common signs (5). Usually the most constant finding of uterine rupture is prolonged fetal bradycardia. In Bujold *et al* study, the first finding with these patients was fetal bradycardia in 87% of cases and abdominal pain only was about 5% as the first sign (13(. it was shown that vaginal bleeding is occurred in 11-67% of cases and maternal shock due to hypervolemia in 29-46% of cases is seen (14, 15). Our patient in sonography had FHR (fetal heart rate) about 60 beat/min.

The myomectomy of our patient in 2009 was performed which 19 myoma were extracted throughout the uterus and inner layer of uterus was repaired. Because of this situation, it was mentioned that if vaginal bleeding or pain occurs during pregnancy, she must return to hospital immediately. Factors affecting uterine rupture after myomectomy include surgery techniques and incision repair, infection and hematoma leading to poor healing of uterine scar. Uterine rupture is not a common complication needed to emergency cesarean section. It is more common that occurring rupture without any remarkable feto-maternal complications. In dehiscence uterine serosa remains safe and no bleeding is happened. The results of some studies revealed that if the duration between 2 pregnancies is shorter that 18 months, it is a risk factor for rupture (16). The sonography has very important role for rupture diagnosis after previous scar (5). The most constant finding is fetal bradycardia.

Uterine rupture is an acute process and diagnosis is very important to save fetus from irreversible damages. In patient with rupture potential, we can use various ways for diagnosis such as amniography and radiopelvimetry, and pelvic exam that is not appropriate for patient with trend to cesarean section. Some studies suggest abdominal & vaginal sonography and sonohystography for diagnosis (17, 18). CT & MRI are not very helpful but MRI is better than CT (17, 18). In our case, fetal bradycardia was present. In Moniha *et al* study also 6 fetuses of 11 birth had bradycardia and 91% had acidosis in Bujold study 2 neonates of 23, had hypoxic ischemic encephalopathy even though emergency intervention (13, 19). Neonatal death incidence is high. Leung et al showed that for 99 cases, 6 neonatal deaths were occurred also Landon *et al* reported that in an academic center in USA, from 124 patients, 2 cases had neonatal death (2%) (20). Although this incidence is decreased in developed countries (21).

Maternal symptoms and signs include massive bleeding developing to hypovolemic shock, bladder damage, hysterectomy and finally maternal death. In our case with corneal rupture in fundal part of uterus, saving uterus was possible with no bladder damage. She just had sign of internal bleeding and anemia without circulatory shock. In Leung et al study in 99 patients with uterine rupture, one case of maternal death was reported (1%) (22). So a law golden time for successful intervention is about 10-37 min after diagnosing and delivery must be done by normal vaginal delivery or cesarean section as soon as possible (19-23).

## Conclusion

In patient with history of previous uterus surgery and abdominal pain uterine rupture must be suspected, even in early pregnancy. First sign in prolonged fetus bradycardia. Early diagnosis of uterine rupture after myomectomy can save patients from death.
